# Human iPSCs-Derived Endothelial Cells with Mutation in *HNF1A* as a Model of Maturity-Onset Diabetes of the Young

**DOI:** 10.3390/cells8111440

**Published:** 2019-11-14

**Authors:** Neli Kachamakova-Trojanowska, Jacek Stepniewski, Jozef Dulak

**Affiliations:** 1 Malopolska Centre of Biotechnology, Jagiellonian University, 30-387 Krakow, Poland; jozef.dulak@uj.edu.pl; 2 Department of Medical Biotechnology, Faculty of Biochemistry, Biophysics and Biotechnology, Jagiellonian University, 30-387 Krakow, Poland; jacek.stepniewski@uj.edu.pl; 3 Kardio-Med Silesia, 41-800 Zabrze, Poland

**Keywords:** iPSCs, endothelial cells, maturity-onset diabetes of the young (MODY), disease modeling, HNF1A, CRISPR/Cas9, genetic background

## Abstract

Patients with *HNF1A*-maturity-onset diabetes of the young (MODY) often develop endothelial dysfunction and related microvascular complications, like retinopathy. As the clinical phenotype of *HNF1A*-MODY diabetes varies considerably, we used human induced pluripotent stem cells (hiPSCs) from two healthy individuals (control) to generate isogenic lines with mutation in *HNF1A* gene. Subsequently, control hiPSCs and their respective *HNF1A* clones were differentiated toward endothelial cells (hiPSC-ECs) and different markers/functions were compared. Human iPSC-ECs from all cell lines showed similar expression of CD31 and Tie-2. VE-cadherin expression was lower in *HNF1A*-mutated isogenic lines, but only in clones derived from one control hiPSCs. In the other isogenic set and cells derived from *HNF1A*-MODY patients, no difference in VE-cadherin expression was observed, suggesting the impact of the genetic background on this endothelial marker. All tested hiPSC-ECs showed an expected angiogenic response regardless of the mutation introduced. Isogenic hiPSC-ECs responded similarly to stimulation with pro-inflammatory cytokine TNF-α with the increase in ICAM-1 and permeability, however, *HNF1A* mutated hiPSC-ECs showed higher permeability in comparison to the control cells. Summarizing, both mono- and biallelic mutations of *HNF1A* in hiPSC-ECs lead to increased permeability in response to TNF-α in normal glycemic conditions, which may have relevance to *HNF1A*-MODY microvascular complications.

## 1. Introduction

Diabetes is not only a metabolic disease, but is also considered as a vascular disease, due to its effect on macro and microcirculation in many vascular beds. The link between diabetes and an increased incidence of cardiovascular diseases is well established, however recently endothelial dysfunction (ED) was shown to precede the clinical diagnosis of type 2 diabetes (T2D) by several years [1]. Despite many proposed mechanisms for this relationship, the definitive pathogenesis remains unclear, possibly because diabetes patients usually display multiple homeostatic imbalances alongside the typically described hyperglycemia. Such disturbances induce ED independently of the presence of diabetes, indicating a multifactorial etiology rather than hyperglycemia per se [2]. The mechanism of ED may point at new management strategies for the prevention of cardiovascular disease in diabetes [1].

Monogenic forms of diabetes are invaluable "human models" that have contributed to our understanding of the pathophysiological basis of common type 1 diabetes (T1D) and T2D [3]. However, research data on endothelial function in monogenic diabetes, such as maturity-onset diabetes of the young (MODY), are so far very limited [4]. MODY is an autosomal dominant monogenic diabetic disease typically affecting individuals before the age of 25 [5] and closely resembling T2D [6]. MODY constitutes a heterogeneous group of relatively frequent autosomal single-gene disorders. Mutations in the glucokinase (*GCK*) gene, along with hepatocyte nuclear factor 1A (*HNF1A*) gene, are the most frequent causes of MODY [4]. Microvascular complications, particularly retinopathy, seem to be common in *HNF1A*-MODY patients [7]. Despite a favorable lipid profile and decreased C-reactive protein (CRP) level described in these patients, they can be characterized by an increased risk of cardiovascular complications as compared with non-diabetic family members [8]. Therefore, these complications seem to be mainly related to overall hyperglycemic exposure, rather than other non-glycemic factors. However, there are studies describing polymorphism in *HNF1A* locus as a risk factor for cardiovascular diseases [9,10]. The clinical expression in *HNF1A*-MODY patients is highly variable from one family to another or even within the same family. *HNF1A* mutation carriers may be normoglycemic, whereas their siblings may be hyperglycemic [11]. Therefore, it is not clear whether the ED found in *HNF1A*-MODY patients [4] could be due to the diabetes complications or is rather genetic consequence. It was recently shown that *HNF1A*-MODY patients have abnormalities in endothelial function or/and presence of an early atherosclerotic phenotype [4]. Up to date, there are no direct connections between ED and abnormal gene function in one of the most prevalent subtypes of MODY.

Induced pluripotent stem cells (iPSCs) provide a well-defined source of tissue-specific cells and are invaluable disease modeling tools. As *HNF1A*-MODY patients were shown to exhibit diabetic microvascular complications [5], their iPSCs can be used to derive endothelial cells (ECs) and investigate possible mechanisms contributing to the complications. However, the clinical phenotype of *HNF1A*-MODY diabetes varies considerably [12], and studies examining correlations between genotype and phenotype are still rare. Therefore, in the current study, we looked for possible ED using iPSCs as disease modeling tools. *HNF1A*-MODY phenotype was modeled through the introduction of mutations in *HNF1A* gene in two control human induced pluripotent stem cells (hiPSCs) lines, using CRISPR/Cas9. Subsequently, all lines were differentiated toward ECs (hiPSC-ECs) and the phenotype of the derived cells was compared. There was no difference in the expression of key ECs markers like PECAM-1 (CD31), Tie-2, angiopoietins, von Willebrand Factor or endothelial nitric oxide synthase (eNOS). However, in one of the control hiPSC-ECs and its respective mutated lines, a clear difference in the expression level of VE-cadherin was found. This could not be confirmed with another control line, suggesting that this effect is dependent on the genetic background of the iPSCs. Importantly, differentiated cells responded appropriately to angiogenic stimuli independently of *HNF1A* mutation. The same was true regarding ICAM-1 expression in response to pro-inflammatory cytokine tumor necrosis factor alpha (TNF-α). However, hiPSC-ECs with *HNF1A* mutation showed increased permeability after stimulation with TNF-α, which may have relevance to microvascular complications in *HNF1A*-MODY diabetes [7].

## 2. Materials and Methods

### 2.1. Cells and Materials Used

Human-induced pluripotent stem cell line HPSI1013i-kuxp_1 (HipSci) was grown on geltrex-coated 6-well plates in E8 medium (ThermoFisher Scientific, Waltham, MA, USA). Control 2 hiPSCs line was generated from peripheral blood mononuclear cells (PBMCs) collected from healthy volunteers after obtaining informed consent (agreement from Jagiellonian University Bioethical Committee no. 122.6120.303.2016). PBMCs were reprogrammed using non-integrating Sendai vectors (Cytotune-iPS 2.0 Sendai Reprogramming kit, ThermoFisher Scientific, Waltham, MA, USA) according to the manufacturer’s protocol (PBMC feeder-free system). MODY3a and MODY3b hiPSCs lines were generated from fibroblasts isolated from two *HNF1A*-MODY patients as previously described [13] after obtaining informed consent (agreement from Jagiellonian University Bioethical Committee no. KBET/43/B/2012). As in the case of PBMCs, cells were reprogrammed using non-integrating Sendai vectors according to the manufacturer’s protocol (fibroblasts, feeder-free system). After approximately three weeks, hiPSCs colonies were picked and expanded. The pluripotency of all generated hiPSCs lines was confirmed before using the cells for further experiments (Supplementary Figure S1). hiPSCs were cultured on geltrex-coated plates in an E8 medium. All cells were cultured at 37 °C in a humidified incubator with 5% CO^2^ (NuAire, Plymouth, MN, USA). TNF-α was purchased from Shenandoah Biotechnology.

### 2.2. Derivation of Mutant hiPSCs

HPSI line was used to generate *HNF1A*-mutated lines using CRISPR/Cas9 gene-editing method. For that purpose 5 × 10^5^ cells were nucleofected with pLentiCRISPR v2-HNF1A-sgRNA1 (sgRNA sequence: GTACGTCCGCAAGCAGCGAG; GenScript, Piscataway, NJ, USA) using Human Stem Cell Nucleofector Kit 1 (Lonza) and program A023 on Nucleofector 2b device (Lonza, Basel, Switzerland) [14] The used gRNA sequence was designed to uniquely target human *HNF1A* gene and was additionally validated [15]. Similar strategy was applied for CIRSPR/Cas9-mediated gene editing of *HNF1A* exon 2 in Control 2 hiPSCs line. To decrease the possibility of observing off-target effects due to CRISPR/Cas9 modification, a different sgRNA was designed and applied for Control 2 hiPSCs (sequence: CGGGAGGTGGTCGATACCAC). In this case, appropriate oligos (5′-CACCGCGGGAGGTGGTCGATACCAC-3′ and 5′- AAACGTGGTATCGACCACCTCCCGC-3′) were annealed cloned into pSpCas9(BB)-2A-Puro plasmid (Addgene #62988 [16], Watertown, MA, USA) digested with BbsI restriction enzyme. Obtained plasmid was amplified and isolated with the Plasmid Midi AX kit (A&A Biotechnology, Gdynia, Poland) according to the manufacturer’s protocol.

After nucleofection with the appropriate vectors, hiPSCs were seeded on a geltrex-coated well of 12-well plate in the E8 medium supplemented with 10 µM ROCK inhibitor (Abcam, Cambridge, UK). After 24h, the medium was replaced with fresh E8 supplemented with 0.5 µg/mL puromycin (Sigma-Aldrich, St. Louis, Mo, USA) to select cells containing delivered plasmid. Subsequently, the selecting medium was changed with fresh E8 after 24 h and hiPSCs were further cultured until reaching approximately 80 % confluency. To obtain single cell-derived clones, nucleofected cells were then harvested, counted and seeded on a geltrex-coated 10 cm plate (500 cells/plate) in E8 medium supplemented with 10 µM ROCK inhibitor. After approximately 14 days clones were large enough to be picked and further expanded.

To assess the presence of mutations in *HNF1A* locus and to verify whether these mutations were introduced as monoallelic or biallelic, Guide it Genotype Confirmation Kit (TaKaRa, Kusatsu, Japan) was used in the next step was used for HPSI cell line-derived clones. For that purpose, DNA was isolated from each derived hiPSCs clone using Genomic Mini kit (A&A Biotechnology) according to manufacturer’s instruction and subsequently subjected to PCR amplifying the region of *HNF1A* gene containing putative mutations (primer forward: CCTTTATCTGTTCCAGTGTCTGT; primer reverse: CAGGACCAAGTCTACTCCCGTC). PCR product solution was then incubated for 1h at 37 °C with recombinant Cas9 nuclease bound to in vitro transcribed sgRNA sequence (the same as in the pLentiCRISPR v2-HNF1-sgRNA1 plasmid, prepared with Guide-it sgRNA In Vitro Transcription kit according to the company’s protocol). Upon inactivation of Cas9 nuclease (5 min, 80 °C), the samples were run on 2% agarose gel. All steps were performed according to the instruction provided to the Guide it Genotype Confirmation Kit. To confirm the presence of mutations in selected hiPSCs clones from both control lines, Surveyor nuclease assay was further performed. For that purpose, isolated DNA was subjected to PCR using the KAPA2G Fast Genotyping Mix (Sigma-Aldrich, St. Louis, Mo, USA) and the same pair of primers as in Guide it Genotype Confirmation Kit. Subsequently, 9 µL of PCR product solution was mixed with 1,5 µL CEL I buffer (custom made at the Department of Cell Biochemistry, Faculty of Biochemistry, Biophysics and Biotechnology, Jagiellonian University, Krakow, Poland) and subjected to heteroduplex formation upon heating to 95 °C and gradual decreasing the temperature to 25 °C. After heteroduplex formation, 1 µL of Celery Juice Extract (CJE, custom made at the Department of Cell Biochemistry, Faculty of Biochemistry, Biophysics and Biotechnology, Jagiellonian University, Krakow, Poland) and 3.5 µL of H_2_O was added and samples were incubated at 45 °C for 45 min and run on 2% agarose gel. To assess the type of mutation introduced in the two selected HPSI and Control 2-derived hiPSC clones, an amplified *HNF1A* region containing mutations was sent for sequencing analysis (Genomed, Warsaw, Poland).

### 2.3. Differentiation toward Endothelial Cells (ECs)

**Scheme 1:**
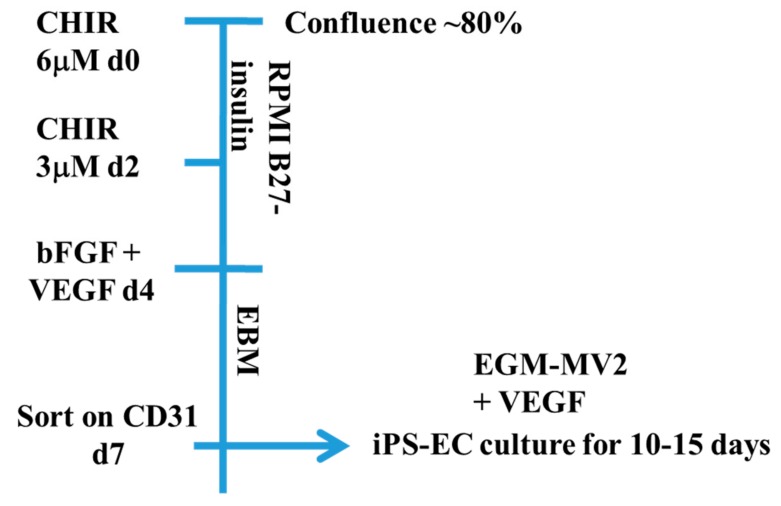
Schematic representation of the protocol used for endothelial differentiation of the human induced pluripotent stem cells (hiPSC)s.

All hiPSCs lines were differentiated based on previously published protocol [17] with modifications. hiPSCs were seeded on geltrex-coated 12-well plates with cell density 1 × 10^5^ cells/well in E8 medium with 10 µM Y-27632 (Focus Biomolecules, Plymouth Meeting, PA, USA). Then the medium was changed daily with E8 alone. On day 4, after reaching around 80–90% confluence, the medium was changed to RPMI supplemented with B-27 without insulin (ThermoFisher Scientific, Waltham, MA, USA) and 6 μM CHIR-99021 (Focus Biomolecules, Plymouth Meeting, PA, USA). Two days later the medium was changed to RPMI supplemented with B-27 without insulin and 3 μM CHIR-99021 and kept for 2 days when the medium was replaced by endothelial basic medium (Promocell, Heidelberg, Germany) with 50 ng/mL VEGF (StemCell Technology, Vancouver, Canada) and 10 ng/mL bFGF (StemCell Technology, Vancouver, Canada). On day 7 cells were detached with Accutase (Biowest, Nuaille, France) and stained with CD31 (clone WM59, Biolegend, San Diego, CA, USA) and CD144 (clone 55-7H1, BD Biosciences, Franklin Lakes, NJ, USA). After washing the excess antibodies, CD31 positive cells were sorted using MoFlo cell sorter (Beckman Coulter, Brea, CA, USA) and cultured on fibronectin-coated wells in endothelial growth medium (EGM MV2, Promocell, Heidelberg, Germany) with the addition of 15 ng/mL VEGF – complete medium. Differentiated cells were used for experiments in the first two weeks after sorting. The schematic representation of the differentiation procedure is shown in Scheme 1.

### 2.4. FACS Analysis

The expression of endothelial-specific surface proteins was assessed by flow cytometry. Briefly, cells were detached with Accutase and after washing with PBS cells were stained with CD31, CD144, Tie-2 (clone 33.1, Biolegend, San Diego, CA, USA), CD54 (ICAM-1, clone HCD54, Biolegend, San Diego, CA, USA) in PBS + 2% FBS for 20 min at 4 °C in dark. Then excess antibodies were washed, and cells were analyzed by flow cytometer BD Fortessa using DIVA software (BD Biosciences, Franklin Lakes, NJ, USA). Dead cells were excluded from the analysis based on DAPI positive staining.

### 2.5. Immunofluorescent Staining

For detection of intracellular as well as extracellular proteins, hiPSCs and iPSC-ECs were first seeded on culture slides (BD Biosciences, Franklin Lakes, NJ, USA), then cultured for 24–72 h in appropriate culture medium. The staining procedure was performed as described previously [18] with the only change of TBS to PBS. All primary and secondary antibodies used in the current study are listed in Table 1. Nuclei were stained with DAPI. Pictures were taken using an LSM 880 Zeiss confocal microscope and analyzed with Zen 2.6 software (Zeiss, Oberkochen, Germany).

### 2.6. RNA Isolation and Real-Time PCR

RNA was isolated from hiPSC-ECs from both control lines and their respective isogenic clones and subsequently reversed transcribed as described earlier [19]. The expression of CDH5 (VE-cadherin) was analyzed using Step One Plus real-time PCR (Applied Biosystems, Foster City, CA, USA) with the addition of SYBR Green PCR Master Mix (SYBR® Green JumpStart™ Taq, Sigma, St. Louis, Mo, USA). The following primer pairs for CHD5 were used: hVE-cad_F: 5′GCACCAGTTTGGCCAATATA3′; hVE-cad_R: 5′GGGTTTTTGCATAATAAGCAGG3′ [20]. The expression of VE-cadherin was normalized to eukaryotic translation elongation factor 2 (EEF2 forward: 5′TCAGCACACTGGCATAGAGG 3′, reverse: 5′GACATCACCAAGGGTGTGCA3′) [19]. Gene expression was calculated based on the comparative CT (threshold cycle value) method (ΔCT = CT gene of interest—CT housekeeping gene). The melting curve analysis was performed immediately after each qPCR for the presence of primer-dimers or non-specific products.

### 2.7. Endothelial Sprouting Assay

The angiogenic sprouting assay was performed as previously described [13]. The basic medium applied to solidified collagen was EBM with 0.5% FBS, whereas the complete medium was EGM MV2 + 15 ng/mL VEGF. The sprouts were counted and measured after 24–48h. The lengths of sprouts were calculated with AxioVision LE64 software (release 4.9.1 Zeiss, Oberkochen, Germany).

### 2.8. Matrigel Tube-Formation Assay

hiPSC-ECs were detached with Accutase and then 10,000 cells were seeded on the top of 50 μL solidified Matrigel (BD Biosciences, Franklin Lakes, NJ, USA) in complete medium (96-well plate) and tube formation was assessed after 16–18 h culture using an inverted microscope.

### 2.9. Permeability Assay

To assess endothelial permeability FITC-dextran permeabilization kit was used (Merck Millipore, Darmstadt, Germany). Briefly, hiPSC-ECs were seeded on collagen-coated transwells for 72h in complete medium. Then medium was gently changed to complete medium or stimulating medium (complete medium + 100 ng/mL TNF-α) for 24 h, followed by incubation with FITC-dextran for 15 min (following manufacturer’s instructions). The intensity of FITC fluorescence connected to the dextran, which passed through the cell monolayer was measured with a Tecan multi-plate reader.

### 2.10. Statistical Analysis

All tests were performed at least in three independent differentiation experiments and statistical significance was tested with t-test or one-way ANOVA (Graphpad, San Diego, CA, USA).

## 3. Results

### 3.1. Derivation of hiPSCs Lines with Mutation in HNF1A Gene and Proving Their Pluripotency

hiPSCs were used previously to study disease mechanisms of ECs [17,21]. One of the critical issues with iPSC-based disease modeling is the use of the respective appropriate control [22]. In the current study, we created isogenic lines by gene editing two hiPSCs lines from healthy individuals, looking for mutation-specific changes unrelated to the individual genetic background. This isogenic iPSCs approach bridges gap in the derivation of disease model from rare diseases with disease-associated mutation, because of its relative ease of use and high efficiency.

**Figure 1:**
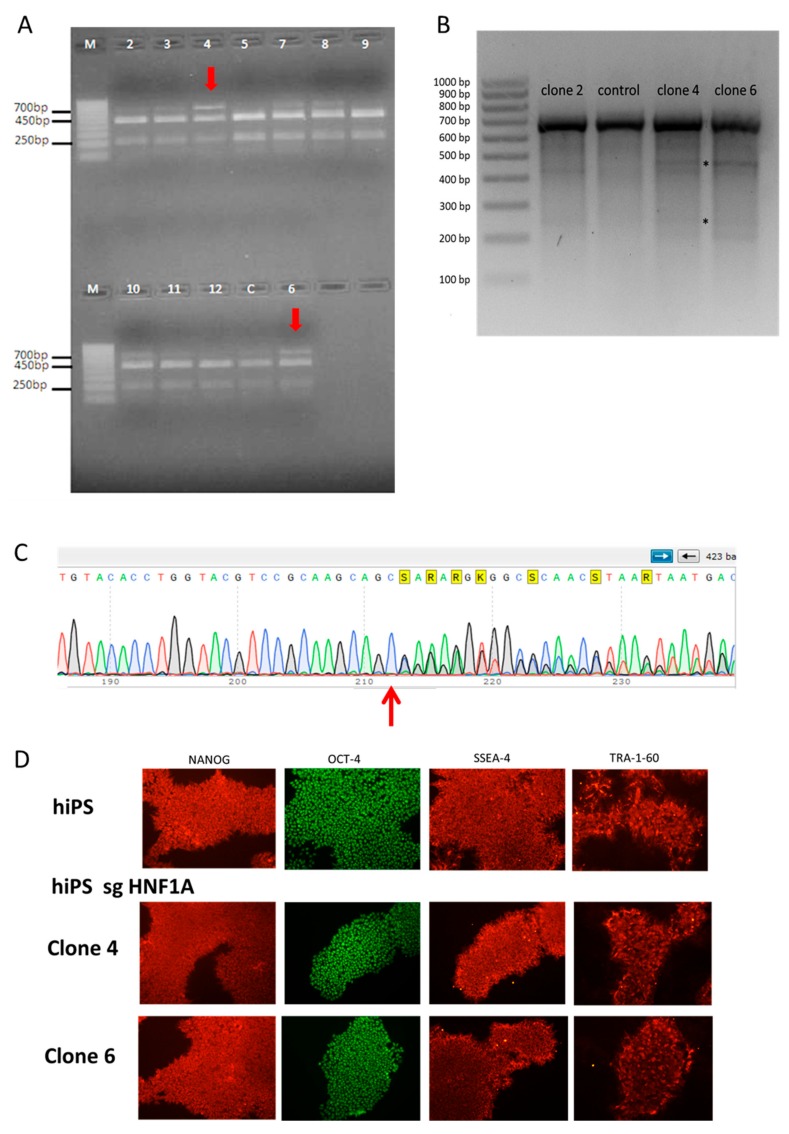
Generation of isogenic human induced pluripotent stem cell (hiPSC) lines; (**A**) results from the Guide-it Genotype Confirmation kit, the red arrows show introduction of monoallelic mutation in HPSI clones; (**B**) results from the surveyor assay of few selected clones, with asterisks showing the cleaved products indicating the presence of heteroduplexes; (**C**) sequencing results from clone 4 (HPSI-derived clone) showing the introduction of the additional cysteine (red arrow), which leads to frameshift and premature stop codon in one of *HNF1A* alleles; (**D**) staining of pluripotency markers (NANOG, OCT-4, SSEA-4 and TRA-1-60) in HPSI control and *HNF1A* mutated hiPSCs lines.

The hiPSCs from HPSI control line were nucleofected with commercially available plasmids encoding Cas9 and sgRNA targeting exon 2 and exon 4 of *HNF1A* gene and then selected with puromycin. The hiPSCs control 2 line, from another healthy individual, was nucleofected with pSpCas9(BB)-P2A-Puro encoding a different sgRNA sequence to additionally limit the risk that observed changes were due to sgRNA-specific off-target effects. After the selection, cells were seeded in lower density to obtain single-cell colonies, which were then expanded and used for isolation of DNA. The introduction of mutations was checked with Guide-it™ Genotype Confirmation Kit (Figure 1A) for HPSI-derived clones and surveyor assay (Figure 1B). Finally, the mutations in the chosen clones from both control hiPSCs lines were confirmed by DNA sequencing (Figure 1C and Figure S1A). In the clones derived from the HPSI line, there was monoallelic insertion of additional cysteine leading to frameshift and premature stop codon (Figure 1C), similar to the mutations occurring in *HNF1A*-MODY patients [23,24]. From the second control line, one clone had a heterozygous mutation with deletion of 13 nucleotides, leading to a premature STOP codon (monoallelic clone, MAC), whereas the second clone had deletion of 13 nucleotides in both alleles of *HNF1A* (biallelic clone, BAC), (Figure S1A). In order to check whether mutagenesis influenced hiPSCs pluripotency, iPSCs were stained for several markers like OCT4, NANOG, SSEA-4, and TRA-1-60. There was no difference in the expression of pluripotency markers between the control lines and the clones with *HNF1A* mutation (Figure 1D, Figure S1B), showing that introduced mutations do not affect hiPSCs function.

The pluripotency of hiPSCs lines derived from two *HNF1A*-MODY patients (MODY3a and MODY3b) were proven as well using the above mentioned pluripotent markers (Figure S1C).

### 3.2. Mutation in HNF1A Does Not Have an Effect on the Expression of Several Endothelial-Specific Markers

**Figure 2:**
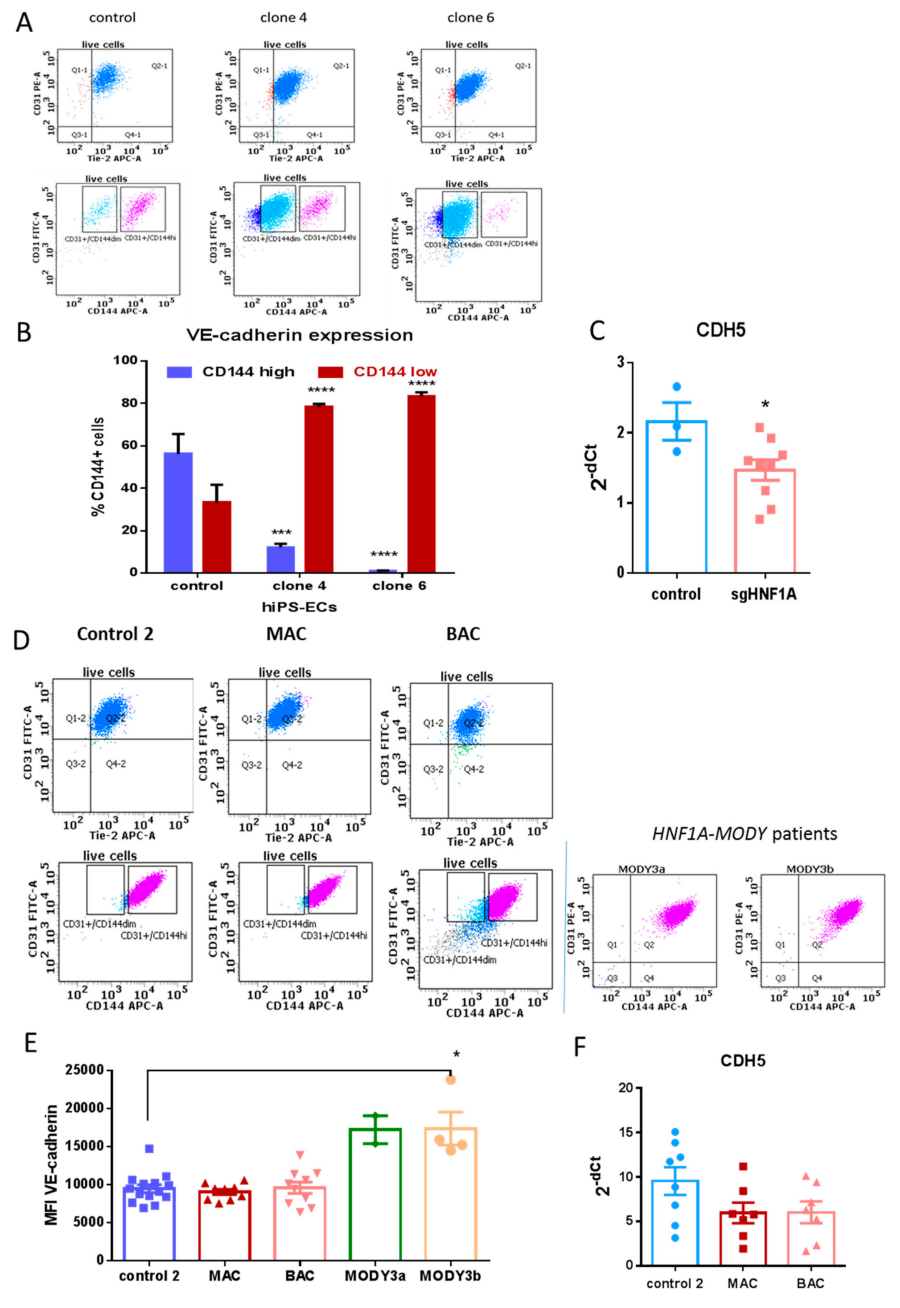
Expression of endothelial markers in hiPSC-endothelial cells (ECs); (**A**) representative dot plots from FACS analysis of CD31, Tie-2 and CD144 expression in control and *HNF1A*-mutated hiPSC-ECs from HPSI control line; The gates in the lower panel show the clear difference in VE-cadherin expression—cells with high and low (dim) expression are marked; (**B**) mean percentage with SEM of two cell populations with high and low VE-cadherin expression in HPSI control line and respective clones, based on FACS analysis, are presented from at least five independent differentiations, *** *p* < 0.0005, **** *p* < 0.0001; (**C**) relative expression of VE-cadherin in hiPSC-ECs from HPSI control and *HNF1A*-mutated lines, analyzed by real-time PCR; results are presented as 2^−ΔCt^ where dots represent means of at least 3 independent differentiation experiments; * *p* < 0.05; (**D**) representative dot plots from FACS analysis of CD31, Tie-2 and CD144 expression in iPSC-ECs from control 2 together with monoallelic (MAC) and biallelic mutated clones (BAC). In the right panel the expression of CD144 and CD31 of iPS-ECs from two *HNF1A*-MODY patients is presented; (**E**) median fluorescence intensity of CD144 (VE-cadherin) of hiPSC-ECs from control 2 and its respective isogenic mutated lines – MAC (monoallelic clone) and BAC, together with hiPSC-ECs from two *HNF1A*-MODY patients; * *p* < 0.05; (**F**). Relative expression of VE-cadherin in hiPSC-ECs from control 2 and the isogenic clones MAC, and BAC, analyzed by real-time PCR; results are presented as 2^−ΔCt^. No statistical difference between the isogenic lines was found.

Both control and mutated hiPSCs were successfully differentiated toward endothelial cells (hiPSC-ECs), although the differentiation efficiency was different for the two control hiPSCs lines. Higher efficiency was observed for hiPSCs control 2 line in comparison to the HPSI one (results not shown), but no effect of *HNF1A* mutation on differentiation efficiency was found. hiPSC-ECs from all lines expressed typical endothelial markers like PECAM-1 (CD31), CD144 (VE-cadherin) and Tie-2 (Figure 2A,D). There was no difference in the expression levels of CD31 and Tie-2. Regarding VE-cadherin expression, in HPSI lines two populations were clearly visible—with high and low expression of the protein. Most of the cells with mutation in *HNF1A* showed lower expression of VE-cadherin as compared to the control iPSC-ECs (Figure 2B). This decrease of VE-cadherin in sgHNF1A lines was also visible on mRNA level (Figure 2C). However, hiPSC-ECs derived from the control 2 line, had only one population with high VE-cadherin expression, which was not altered by the monoallelic or biallelic mutation in *HNF1A* (Figure 2D,E). This lack of difference was confirmed also on mRNA level (Figure 2F). Moreover, in hiPSC-ECs from two *HNF1A*-MODY patients, there was also no difference in VE-cadherin expression (Figure 2D,E). Even a slight increase in the median fluorescence intensity (MFI) in one of the *HNF1A*-MODY patients in comparison to control 2 was found (Figure 2E). This strongly suggests, that the effect observed for VE-cadherin in HPSI line could be attributed to the genetic background of this control line, which in combination with *HNF1A* mutation further impairs VE-cadherin expression.

Other proteins like phospho-eNOS, von Willebrand factor, angiopoietin 1 and 2 were similarly present in hiPSC-ECs from all lines (Figure S2A–D). Additionally, no visible change in VE-cadherin localization between the hiPSC-ECs lines could be seen.

### 3.3. HNF1A Mutation Does Not Cause Differences in Angiogenic Capacity of Differentiated hiPSC-ECs

**Figure 3:**
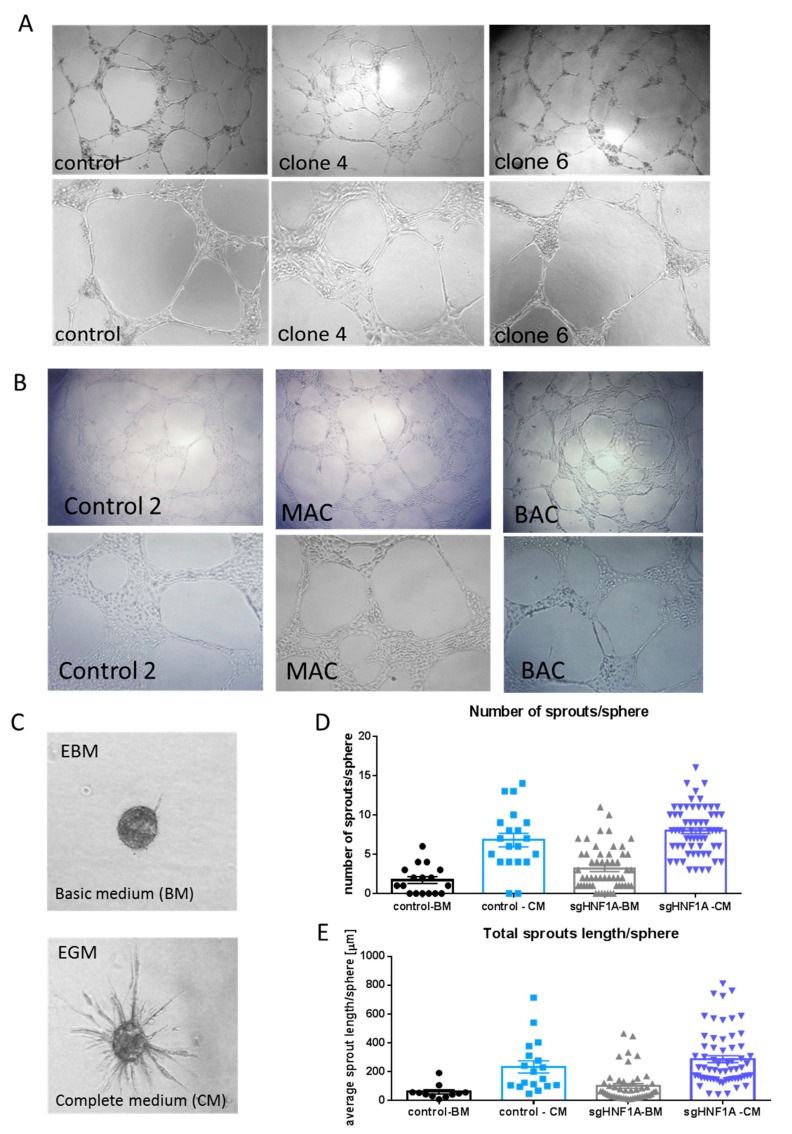
Angiogenic capacity of hiPSC-ECs; (**A**) representative pictures of Matrigel tube-formation assay of cells with heterozygous mutation in *HNF1A* gene together with the respective HPSI control cells (upper panel—magnification 40×, lower panel—agnification 100×); (**B**) representative pictures of Matrigel tube-formation assay of cells with monoallelic (MAC) and biallelic (BAC) mutation in *HNF1A* gene with the respective control 2 cells (upper panel—magnification 40×, lower panel—magnification 100×); (**C**) representative pictures of spheres formed by hiPSC-ECs in basic medium (BM) and in complete medium (CM); (**D**) calculation of the mean number of sprouts/sphere (±SEM); (**E**) total sprout length per sphere (±SEM). For (**D**) and (**E**) summarized data from both clone 4 and clone 6 are presented as sgHNF1A. No statistical difference in angiogenic capacity between the lines could be observed.

Apart from hyperglycemia, abnormalities of angiogenesis may cause or contribute toward many of the clinical manifestations of diabetes. The main technical challenge in angiogenesis studies is the selection of the most appropriate assay. As there is no “gold-standard” angiogenic assay, a combination of assays is required [25]. The angiogenic capacity of differentiated cells was checked with two commonly used in vitro assays. The tube-formation capacity of differentiated cells was independent of the genetic background of the control line or the mutations in *HNF1A* gene (Figure 3A,B). Additionally, the response of differentiated cells to angiogenic stimuli (like VEGF) was tested with endothelial sprouting assay. hiPSC-ECs with and without mutation in *HNF1A* formed sprouts in the 3D collagen matrix in response to angiogenic stimuli, which are included in the complete cell culture medium, whereas no or less sprouts were observed when cells were cultured in basic medium (without growth factors and lower FBS concentration, Figure 3C). There was no difference in the endothelial sprouting assay response between the isogenic lines derived from HPSI hiPSCs line, both in basic and complete medium, as measured by average sprout number/sphere (Figure 3D) and total sprout length/sphere (Figure 3E). The results from both assays showed that mutations in *HNF1A* does not have an impact on endothelial angiogenic capacity.

### 3.4. Differentiated Cells Respond Appropriately to Pro-Inflammatory Cytokine with No Effect of HNF1A Mutation

**Figure 4:**
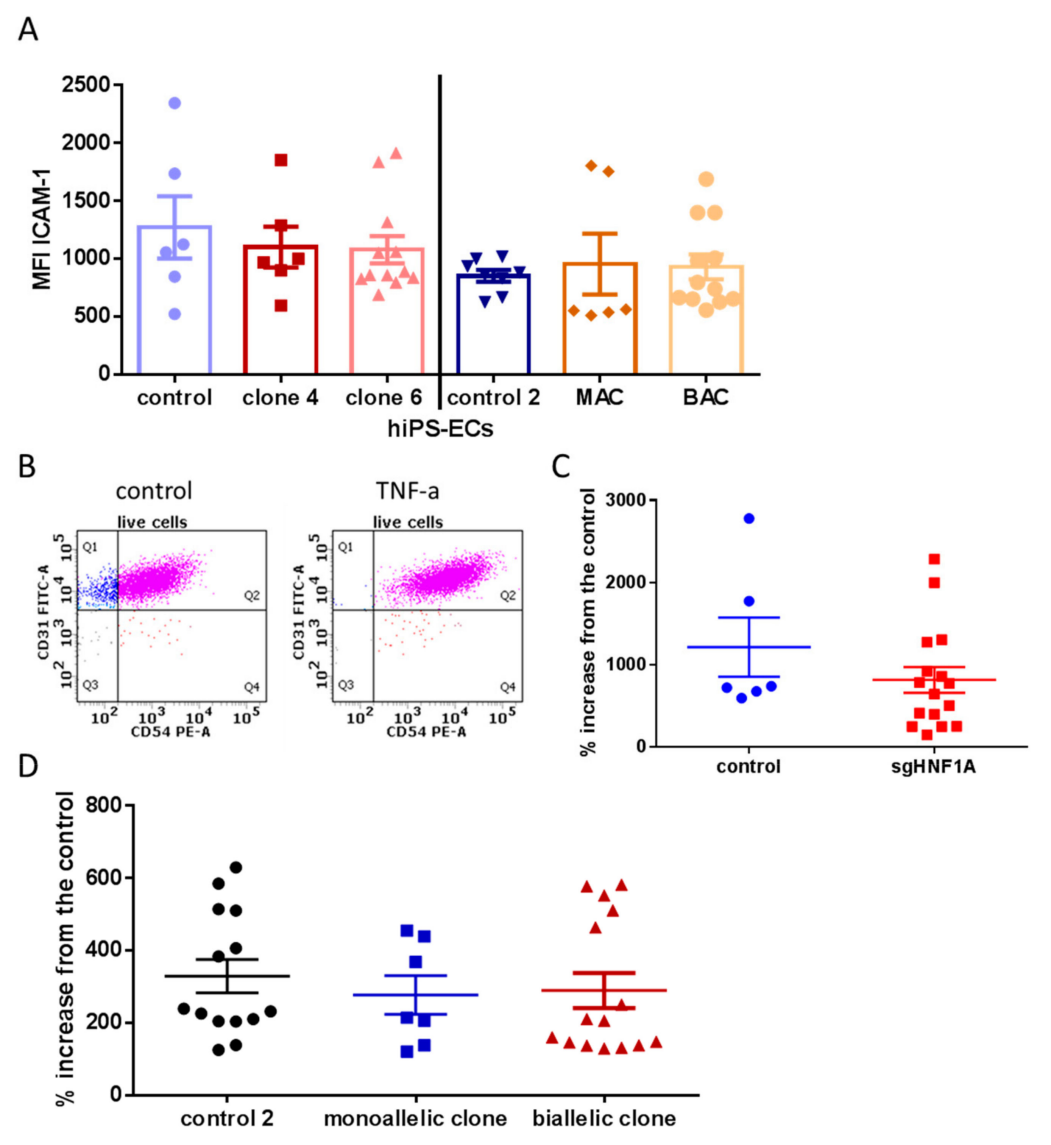
Response to pro-inflammatory cytokine (TNFα); (**A**) median fluorescence intensity (MFI) of ICAM-1 in hiPSC-ECs cultured in basic conditions (complete medium)—mean ± SEM, data is taken from at least 3 independent differentiation experiments; (**B**) representative dot plot; (**C**) increase in ICAM-1 expression presented as percentage from respective control cells (cells not treated with the cytokine), showing no difference in ICAM-1 increase between the normal hiPSC-ECs and cells with introduced mutation in *HNF1A* gene (sgHNF1A shows summarized data from both clones); (**D**) increase in ICAM-1 expression presented as percentage from respective control cells (cells not treated with the cytokine), showing no difference in ICAM-1 increase between the normal hiPSC-ECs (control 2) and cells with monoallelic or biallelic mutation in *HNF1A* gene.

Another feature of the mature endothelium is the ability to respond to pro-inflammatory stimuli and participate in the inflammatory reaction. Under normal physiological conditions, the endothelium is in a quiescent and anti-inflammatory state, however, in the presence of risk factors, it can be activated to express adhesion molecules like intracellular adhesion molecule-1 (ICAM-1) [26]. These molecules are required for communication of the endothelium with the immune system (leukocytes). Additionally, it is known that there is an increase in pro-inflammatory cytokines like TNFα in diabetic retinopathy [27]. Therefore, we checked the expression of ICAM-1 in basic conditions and after stimulation with 20 ng/mL TNFα There was no difference in ICAM-1 expression in basic conditions related to *HNF1A* mutation, seen for both control lines and their respective mutated clones (Figure 4A). All cell lines responded with an appropriate increase in ICAM-1 expression when treated with TNFα (Figure 4B), but no difference in this response in relation to *HNF1A* mutation could be observed (Figure 4C,D).

### 3.5. Increased Permeability of hiPSC-ECs with Mutation in HNF1A After Pro-Inflammatory Stimuli

**Figure 5:**
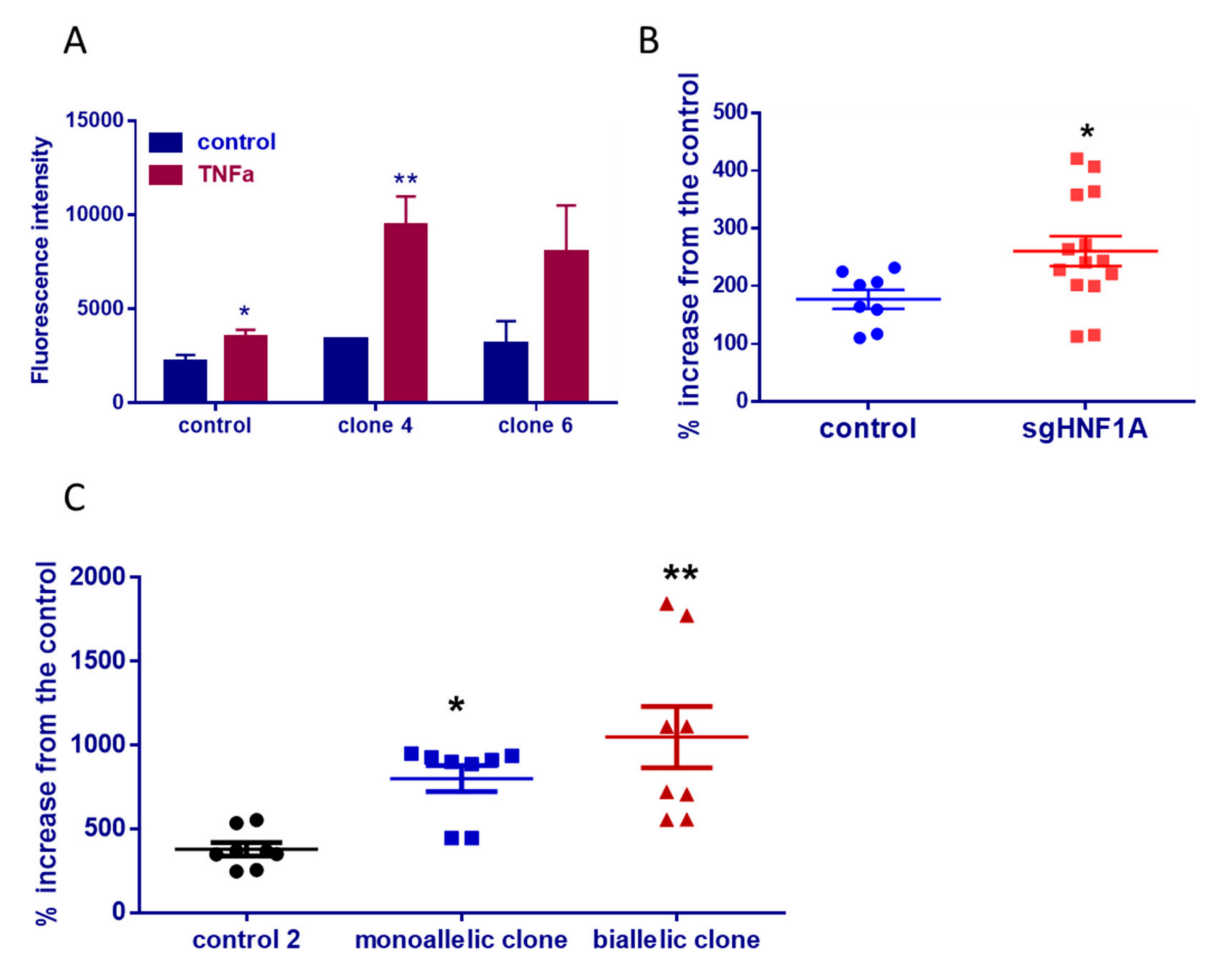
Permeability assay of hiPSC-ECs; (**A**) TNFα causes increase in endothelial permeability as measured by the increase in FITC-dextran fluorescence intensity in all tested lines, data presented as means ±SEM, * *p* < 0.05, ** *p* < 0.01; (**B**) Increased permeability after stimulation with TNFα in hiPSC-ECs with mutation in *HNF1A* in comparison to the non-mutated cells, data is presented as percentage from the respective controls (cells cultured in complete medium without cytokine stimulation), where dots are means ± SEM, * *p* < 0.05; (**C**) Increased permeability after stimulation with TNFα in hiPSC-ECs with monoallelic or biallelic mutation in *HNF1A* in comparison to the non-mutated cells, data are presented as percentage from the respective controls (cells cultured in complete medium without cytokine stimulation), where dots are means ± SEM, * *p* < 0.05; ** *p* < 0.01.

In response to TNFα, ECs respond not only with increased expression in adhesion molecules but also with increased permeability. Additionally, it is known that the consequences of the biochemical changes in diabetic retinopathy, like inflammation and oxidative stress, lead to vascular permeability and capillary rarefaction [28]. Therefore, we looked at the vascular permeability of iPSC-ECs in response to pro-inflammatory cytokine. Results show that in all hiPSC-ECs there was an increase in vascular permeability after TNFα stimulation (Figure 5A). However, the cells with heterozygous mutation in *HNF1A* responded with a much higher increase in vascular permeability (Figure 5B), pointing at certain susceptibility of *HNF1A*-MODY ECs to diabetic complications as diabetic retinopathy. Importantly, the same effect was observed with the second set of isogenic lines (Figure 5C). Moreover, this increase was the highest, when *HNF1A* was mutated in both alleles, pointing at a strong relation between the increased endothelial permeability and *HNF1A* mutations.

## 4. Discussion

As abnormal endothelial physiology may cause wide-spread systemic diseases and also play a crucial role in a variety of human disorders, many studies used hiPSCs as modeling tools regarding ED [29]. It is currently clear that subtle microvascular dysfunctions may precede diabetes and also may predict common clinical complications like retinopathy and nephropathy [30]. Additionally, normal microvascular function is necessary for physiological insulin-mediated glucose disposal and glucose-induced insulin secretion, highlighting that association between ED and hyperglycemia in T2D is bidirectional [30]. Therefore, in the current study, we investigated for subtle changes in endothelial function of cells with *HNF1A* mutation, looking for possible pathological readout connected to diabetic retinopathy, as it is a common complication in patients with *HNF1A*-MODY. Using isogenic cell lines to limit the effect of the genetic background [31] we checked the expression of endothelial markers and focused on cells’ responses to pro-angiogenic and pro-inflammatory stimulations.

Multiple protocols have been established for differentiation of iPSCs into ECs [17,32,33,34], although still efforts are made to make the methods more reproducible and with higher yield [29]. Most of the published protocols differ in efficiency, cost, complexity, and variability, therefore commonly the proper choice of EC differentiation protocol depends on the downstream application [35]. In the current study the modified protocol of Gu et al., for EC differentiation was used [17]. Modifying the protocol allowed for the increase of the yield from 1–2% to 20–25 %. Using this protocol for all generated hiPSCs lines, no difference in the differentiation capacity toward endothelium, connected to *HNF1A* mutations, was found.

Differentiated iPSC-ECs showed expression of typical endothelial markers like CD31, Tie-2, angiopoietins (1 and 2) and von Willebrand Factor. PECAM-1 (CD31) is highly expressed at ECs intercellular junctions and has multiple functions like regulation of leukocyte trafficking and maintenance of EC junctional integrity [36]. There was no difference in CD31 expression between ECs with and without *HNF1A* mutation. Angiopoietins (Ang) act through the tyrosine kinase receptor Tie-2, which in turn regulates survival, apoptosis, regulates capillary sprouting or controls vascular permeability [37]. Ang1 is a potent angiogenic growth factor, whereas Ang 2 was considered as antagonist acting through the same receptor, Tie-2. In the current study, no difference in the expression of Ang1 or Tie-2 in hiPSC-ECs with and without *HNF1A* mutation was found. However, recent data points at the more important role of Ang 2 in EC pathophysiology, as its dysregulated expression was found in several disorders like cardiovascular diseases, diabetic retinopathy and obesity [38]. Ang 2 was found to facilitate angiogenesis, vascular permeability and inflammation under certain disease settings [39]. Nevertheless, *HNF1A* mutation did not cause any visible difference in Ang 2 expression in hiPSC-ECs, however additional and more precise evaluation of Ang 2 expression would be needed to confirm the lack of difference in this protein.

VE-cadherin is a very specific endothelial junction molecule with vital importance in maintaining and controlling ECs contacts [40]. It is known that cells modulate their adhesive state by controlling the amount of cadherin localized at the cell surface. The expression of VE-cadherin can be regulated via internalization or by shedding by specific disintegrins and metalloproteinases [41,42]. In previous studies, it was found that patients with T2D have increased levels of plasma VE-cadherin levels [43]. Soluble VE-cadherin can be released in the bloodstream after increased proteolitic activity of ADAM 10 due to inflammation, thus leading to increased permeability [42,44,45]. At this stage, there are no studies showing any connection between soluble VE-cadherin and microvascular complications in *HNF1A*-MODY patients. In the current study, controversial results for VE-cadherin expression were found. The impact of the genetic background of the hiPSCs lines was apparent, as no changes could be observed with cells from one control line together with its respective isogenic mutated clones and in two *HNF1A*-MODY patients. However, when another control hiPSCs line was used, where two distinct populations of cells (with low and high VE-cadherin expression) were present in the control line, a significant decrease in VE-cadherin was found in *HNF1A* mutated clones, both on protein and mRNA levels. Therefore, it could be speculated that the altered VE-cadherin expression, observed in isogenic cell lines derived from the HPSI control line, is a result of the additive impact of the donor genetic background and the *HNF1A* mutation. This indicates that for proper disease modeling of monogenic diseases, where the genetic background could play a significant role together with the mutation in a certain gene, the results from several hiPSCs lines should be taken into consideration.

VE-cadherin participates in multiple aspects of EC biology like maturation, remodeling, and extension of vessels, which are characteristics of angiogenesis [41]. Physiological angiogenesis is a highly organized sequence of cellular events, which are controlled and modulated to meet the tissue requirements. The angiogenesis process is, however, also implicated in the pathogenesis of vascular abnormalities of the retina, kidneys, and fetus, impaired wound healing, increased risk of rejection of transplanted organs, and impaired formation of coronary collaterals. A perplexing feature of the aberrant angiogenesis is that excessive and insufficient angiogenesis can occur in different organs in the same individual [46]. In patients with *HNF1A*-MODY almost every fourth diagnosed individual shows diabetic retinopathy [7], which could be associated with increased VEGF production and neovascularization [47]. In the current study, there was no difference in the angiogenic properties of hiPSC-ECs with and without mutation in the *HNF1A* gene. Using two in vitro tests we show that hiPSC-ECs from all lines respond appropriately to pro-angiogenic factors. In our previous study we have shown that iPSC-EC from murine genetic model of diabetes (db/db mice) have impaired angiogenic capacity in comparison to cells from wild type mice and these findings were confirmed with primary cells isolated from lungs [13], proving that iPSCs-derived ECs can be successfully used as disease modeling tools. Thus, based on our current results, it can be suggested that *HNF1A* mutation does not cause any direct impairment of the angiogenic response of human ECs.

In diabetic retinopathy, there is an increase of pro-inflammatory cytokines and chemokines like monocyte chemoattractant protein 1, TNFα, interleukin 1β (IL-1β) and IL-6 [39]. Therefore, we looked for the hiPSC-ECs response to TNFα. Cells from all lines responded appropriately to pro-inflammatory stimulation with the upregulation of ICAM-1, but this increase was not affected by the introduced *HNF1A* mutation. However, the vascular permeability of ECs with *HNF1A* monoallelic mutation was higher than the permeability of the control cells. In diabetic retinopathy dysfunction of vascular cells in the inner blood-retinal barrier leads to a breakdown of this barrier resulting in vascular leakage and as a consequence vision impairment [28]. A major risk factor for diabetic retinopathy is sustained hyperglycemia, however, certain diabetic populations do not develop such complications, whereas good glycemic control may not eliminate the life-time risk for retinopathy. Altogether, this suggests that there are additional factors, such as genetic susceptibility involved in the initiation and progression of diabetic retinopathy [39].

## 5. Conclusions

In the current study, we have shown for the first time that iPSCs-derived ECs with *HNF1A* mutations have increased permeability in response to TNFα. Importantly, this increase could be detected with both mono- and biallelic mutation clones, independently of the genetic background and as a sole response to pro-inflammatory stimulation. Therefore, it could be suggested that mutations in the *HNF1A* gene, as occurring in *HNF1A*-MODY patients, could predispose for increased vascular permeability and subsequent microvascular complications.

## Figures and Tables

**Table 1:** List of all antibodies used for immunofluorescent stainings.

	Species	Company	Dilution
Von Willebrand Factor	Mouse	ThermoFisher Scientific	1:50
Phospho-eNOS	Rabbit	ThermoFisher Scientific	1:70
VE-cadherin	Mouse	GeneTex	1:500
Angiopoietin 1	Rabbit	Abcam	1:100
Angiopoietin 2	Rabbit	Abcam	1:100
Oct-3/4	Goat	Santa Cruz	1:200
Nanog	Rabbit	Abcam	1:100
SSEA-4	Mouse	Millipore	1:100
TRA-1-60	Mouse	Millipore	1:100
Anti-mouse–AF488, AF568	Goat	Invitrogen	1:1000
Anti-rabbit-AF488, AF568	Goat	Invitrogen	1:1000
Anti-goat AF488	Rabbit	Invitrogen	1:1000

## Supplementary Materials

The following are available online at https://www.mdpi.com/2073-4409/8/11/1440/s1.
