# Supplementary material for: Human iPSCs-Derived Endothelial Cells with Mutation in HNF1A as a Model of Maturity-Onset Diabetes of the Young
Source: Cells. 2019 Nov 14;8(11):1440. doi: 10.3390/cells8111440 (PMC6912300; doi:10.3390/cells8111440)
Supplement: Supplementary file 1 [file cells-08-01440-s001.pdf]

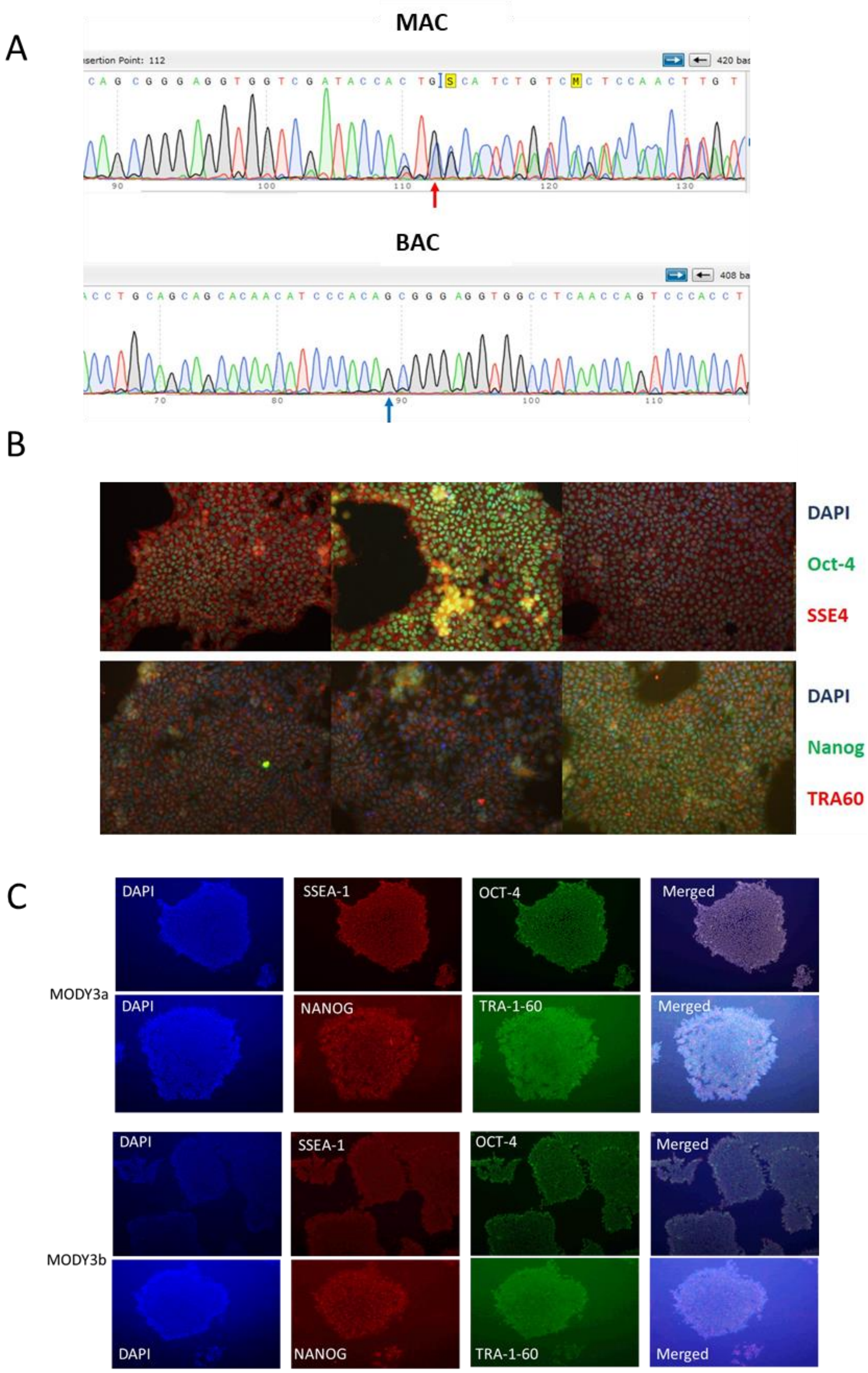

Figure 1. (**A**) Sequencing results from monoallelic clone (MAC) and biallelic clone (BAC) with the arrow indicating place with 13 nucleotide deletion in HNF1A gene; (**B**) Representative pictures of pluripotent markers (OCT4, SSEA-4, NANOG, TRA-1-60) in control 2 and its respective clones; (C) Representative pictures of pluripotent markers of hiPSCs colonies, derived from two *HNF1A*-MODY patients – MODY3a and MODY3b.

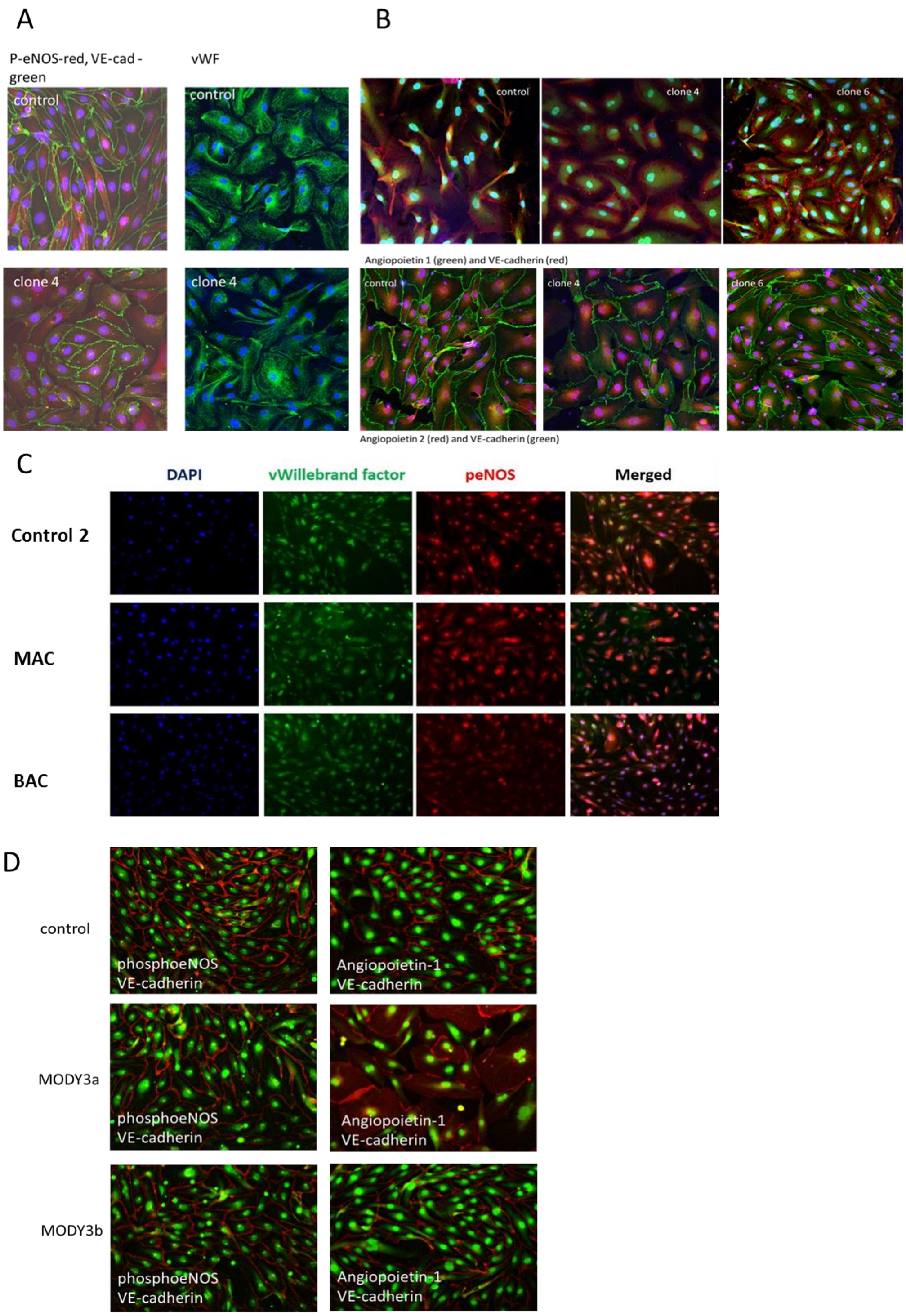

Figure 2. (**A**) Representative pictures showing expression of phospho-eNOS and VE-cadherin (left panel, red-peNOS and green VE-cadherin), von Willebrand Factor (vWF, right panel, green) in HPSI control and *HNF1A* mutated cells (clone 4), in both panels nucleus were stained with DAPI (blue); (**B**) Representative pictures of HPSI control iPSC-ECs and *HNF1A* heterozygous clones, showing expression of angiopoietin 1 (upper panel, green) and angiopoietin 2 (lower panel, red) together with VE-cadherin; (**C**) Representative pictures of vWF (green) and phospho-eNOS (red) in control 2 and respective *HNF1A* clones; (**D**) Representative pictures showing expression of phospho-eNOS (left panel) and angiopoietin 1 (right panel, both in green) together with VE-cadherin (red).
